# Supplementary material for: Comprehensive transcriptomic analysis of COVID-19 blood, lung, and airway
Source: Sci Rep. 2021 Mar 29;11:7052. doi: 10.1038/s41598-021-86002-x (PMC8007747; doi:10.1038/s41598-021-86002-x)
Supplement: Supplementary file 1 — Supplementary Information 1. [file 41598_2021_86002_MOESM1_ESM.pdf]

Supplementary Figures

Comprehensive Transcriptomic Analysis of COVID-19 Blood, Lung, and Airway

Andrea R. Daamen[1,2,*], Prathyusha Bachali[1,2], Katherine A. Owen[1,2], Kathryn M. Kingsmore[1,2], Erika L. Hubbard[1,2], Adam C. Labonte[1], Robert Robl[1], Sneha Shrotri[1], Amrie C. Grammer[1], Peter E. Lipsky[1,3,*]

[1]AMPEL BioSolutions LLC; Charlottesville, Virginia, 22902; United States of America

[2]These authors contributed equally

[3]Lead Contact

*Correspondence: peterlipsky@comcast.net (P.E.L.),

andrea.daamen@ampelbiosolutions.com (A.R.D.)

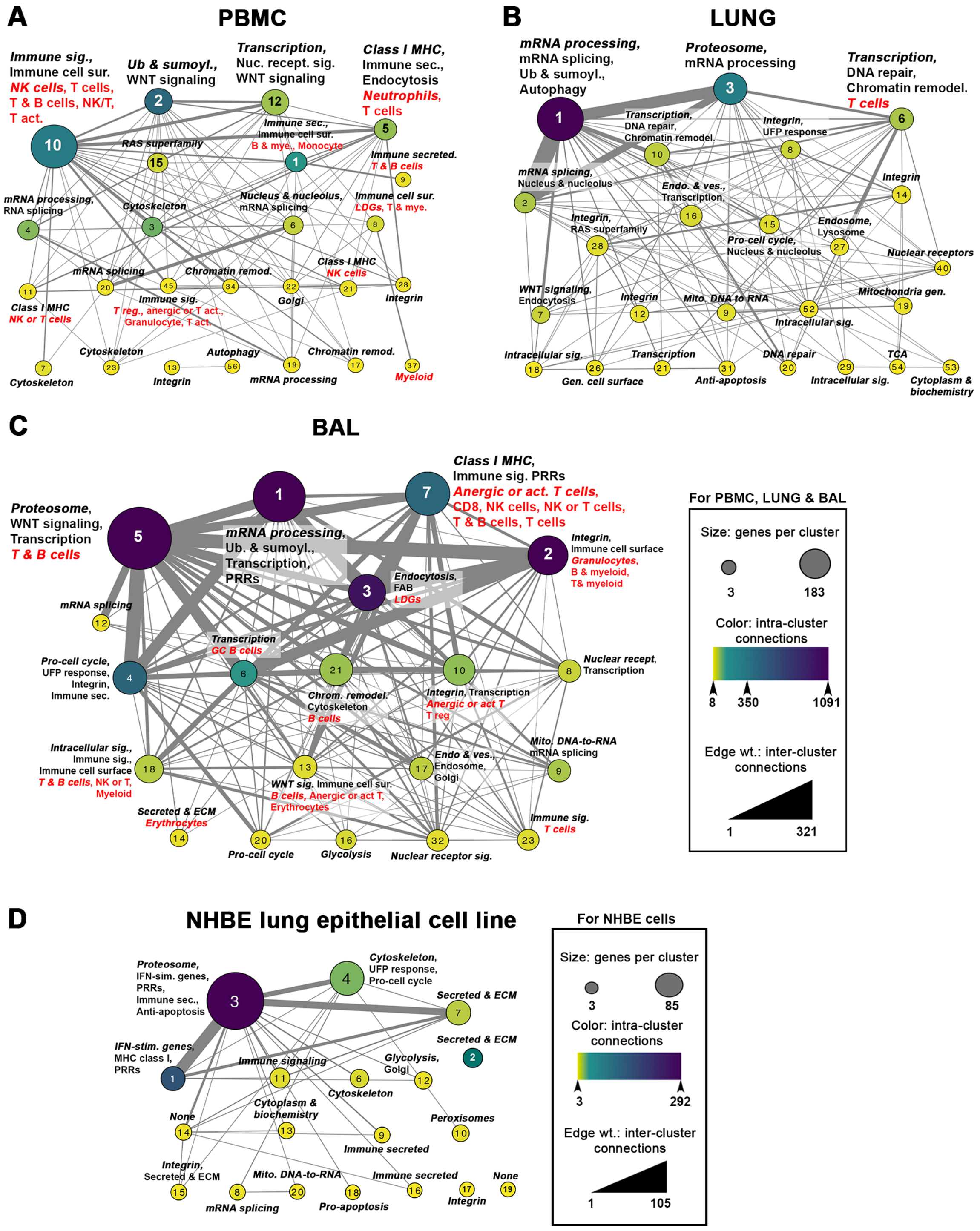

Supplementary Figure 1. Metaclusters identify differentially expressed cell populations and functional gene clusters in SARS-CoV2 infected tissues and cell lines. Down-regulated DE genes from peripheral blood (**A**), lung (**B**) and airway (**C**), and up-regulated DE genes from the NHBE primary lung epithelial cell line (**D**) were used to create metaclusters. Metaclusters were generated based on PPI networks, clustered using MCODE and visualized in Cytoscape as in **Figure 4**. Size indicates the number of genes per cluster, color indicates the number of intra-cluster connections and edge weight indicates the number of inter-cluster connections. Cluster enrichment for biological function and immune cell type was determined by BIG-C and I-Scope, respectively.

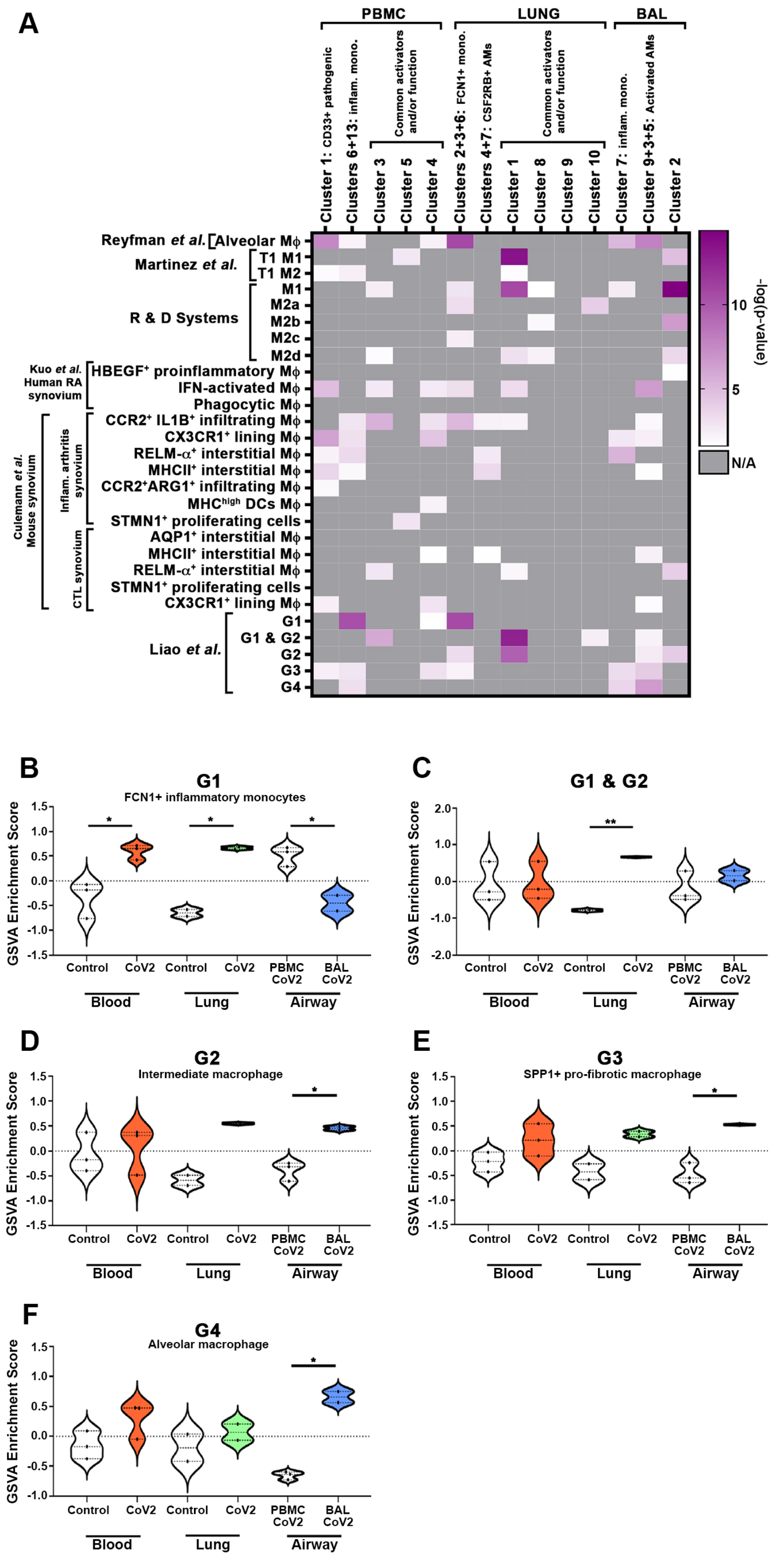

Supplementary Figure 2. Evaluation of published macrophage gene signatures in myeloid-derived clusters from COVID-affected blood, lung and BAL fluid. Previously published macrophage signatures from the indicated sources were compared to myeloid clusters from Figure 3D-F. Heatmap depicts signatures with significant overlap (-log(p-value) >1.33) with myeloid clusters from the blood, lung and airway compartments generated using GraphPad Prism v8.4.2 (www.graphpad.com). N/A, non-applicable/non-significant overlap detected. R & D Systems provided signatures for the M1, M2a, M2b, M2c and M2d populations.

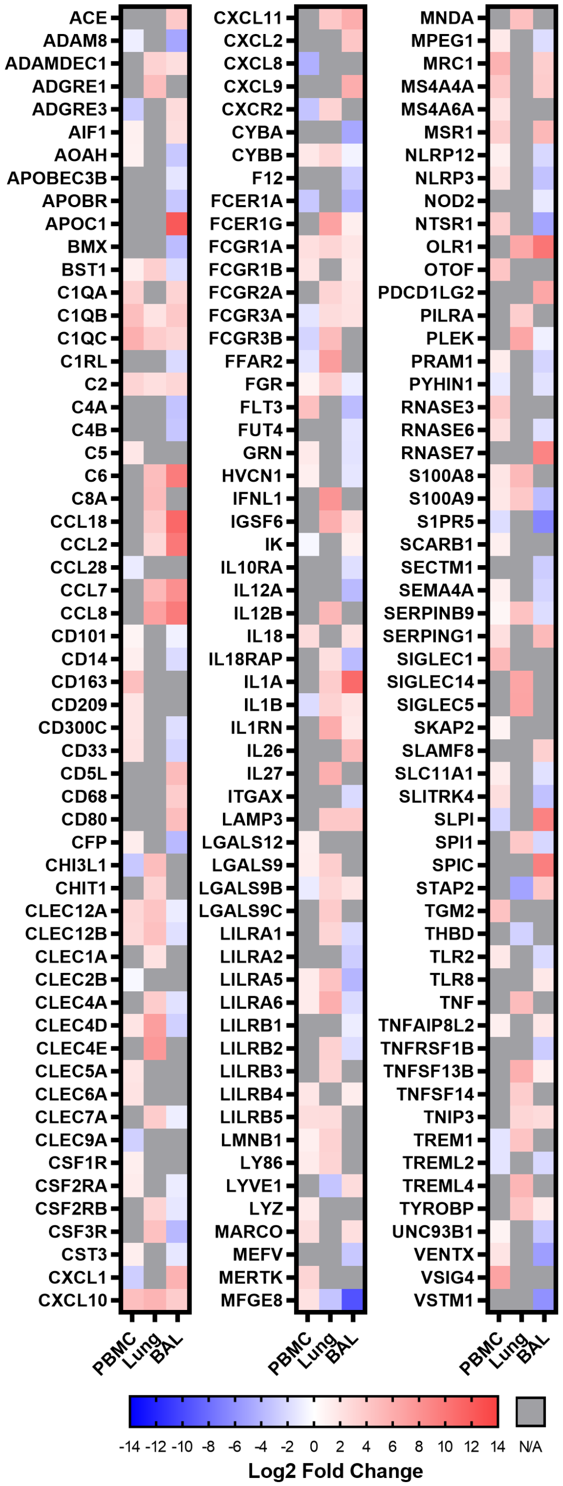

Supplementary Figure 3. Heterogeneous expression of monocyte/myeloid cell genes in different CoV2 tissue compartments as compared to control. Evaluation of differential expression of 171 monocyte/myeloid genes in each compartment reveals shared and disparate expression among the tissues. PBMC represents PBMC-CoV2 to PBMC-CTL. Lung represents Lung-CoV2 to Lung-CTL. BAL represents BAL-CoV2 to PBMC-CoV2. Scale bar presents Log2 Fold Change. N/A represents genes that were not significantly DE at FDR < 0.2. Heatmaps generated using GraphPad Prism v8.4.2 (www.graphpad.com).

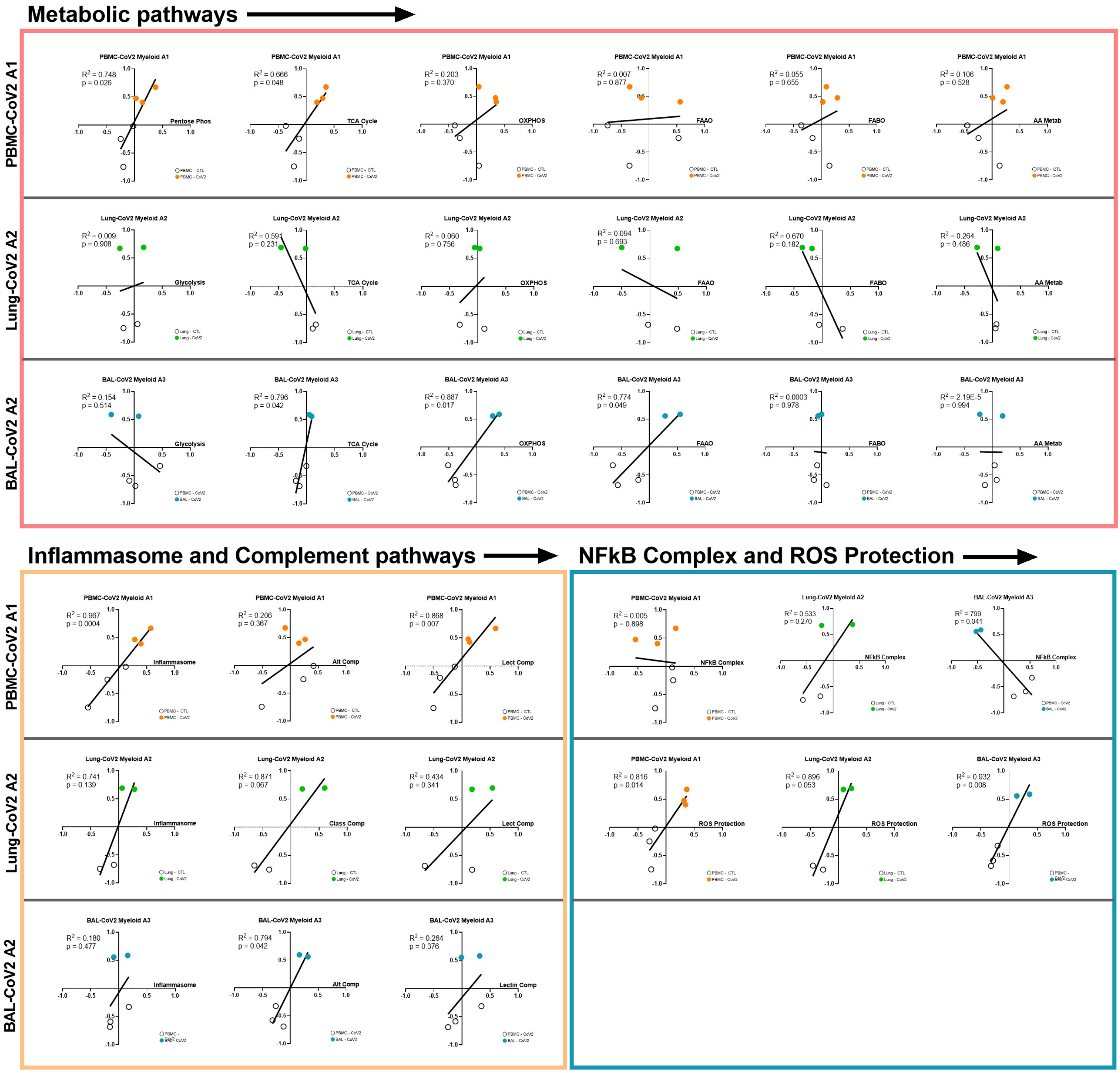

Supplementary Figure 4. Analysis of biological activities of myeloid subpopulations. Linear regression between GSVA scores for each of the tissue-specific myeloid populations and metabolic pathways, inlammasome, complement pathways, NFKB complex signaling and ROS protection. Generated using GraphPad Prism v8.4.2 (www.graphpad.com).

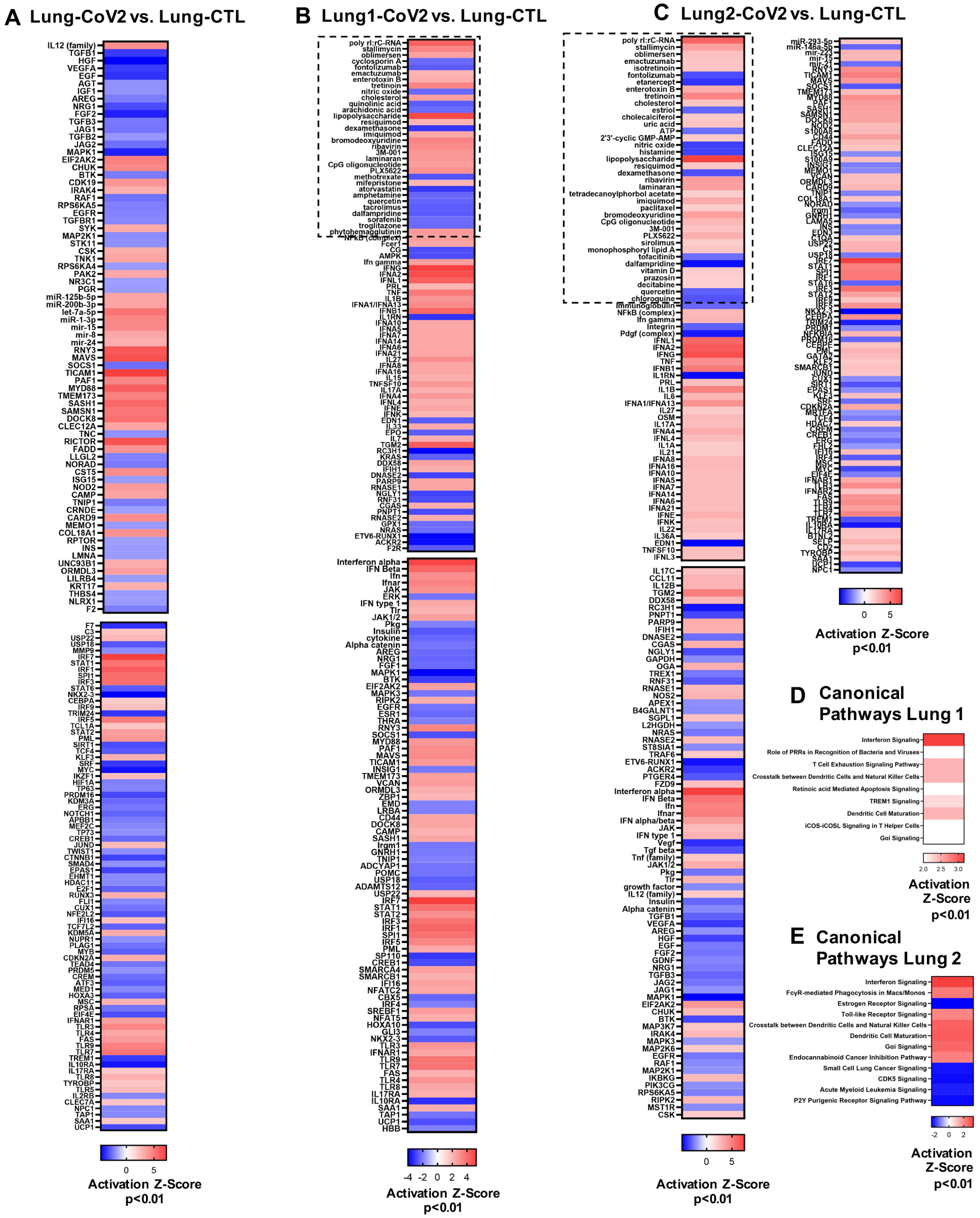

Supplementary Figure 5. Pathway analysis of SARS-CoV-2 lung tissue. (A) Remaining significant upstream regulators operative in SARS-CoV-2 lung tissue predicted by IPA upstream regulator analysis (see Figure 9). Upstream regulator analysis was also conducted on DEGs from each individual COVID-19 lung compared to healthy controls due to observed heterogeneity, with significant results displayed in **(B)** and **(C)**. Chemical reagents, chemical toxicants, and non-mammalian endogenous chemicals were culled from results. The boxes with the dotted outline separate small molecules/drugs/compounds that were predicted as upstream regulators from pathway molecules and complexes. **(D, E)** IPA canonical signaling pathway analysis was conducted on individual COVID-19 lung samples. Pathways and upstream regulators were considered significant by |Activation Z-Score|≥2 and overlap p-value<0.01.
